# Sex-Based Differences in the Myogenic Response and Inflammatory Gene Expression Following Eccentric Contractions in Humans

**DOI:** 10.3389/fphys.2022.880625

**Published:** 2022-04-29

**Authors:** Stephen A. Fortino, Mai Wageh, Riley Pontello, Chris McGlory, Dinesh Kumbhare, Stuart M. Phillips, Gianni Parise

**Affiliations:** ^1^ Department of Kinesiology, McMaster University, Hamilton, ON, Canada; ^2^ School of Kinesiology and Health Studies, Queen’s University, Kingston, ON, Canada; ^3^ Toronto Rehabilitation Institute, University Health Network, Toronto, ON, Canada

**Keywords:** inflammation, myogenesis, regeneration, repair, satellite cell

## Abstract

After muscle injury, the interaction between muscle satellite cells (SC) and the immune response is instrumental for the repair and regeneration of skeletal muscle tissue. Studies have reported sex-based differences in the skeletal muscle inflammatory and regenerative response following injury. However, many of these studies investigated such differences by manipulating the concentration of estradiol, in rodents and humans, without directly comparing males to females. We sought to explore differences in the myogenic and inflammatory response following unaccustomed eccentric exercise in males and females. We hypothesized that females would have a blunted myogenic and inflammatory response as compared to males.

**Methods**: 26 (13 male, 13 female) healthy young adults (22 ± 0.4 years [mean ± SEM]) performed 300 maximal eccentric contractions (180°/s) of the knee extensors. Muscle biopsies were taken before (pre) and 48 h (post) following eccentric damage. SC content and activation were determined by immunohistochemical and real time-polymerase chain reaction (rt-PCR) analysis. Inflammatory markers were analyzed using rt-PCR.

**Results**: Following eccentric damage, males had a greater expansion of type I-associated SC (*p* < 0.05), and there was a trend for a greater expansion in total SC (type I + II fibers) (*p* = 0.06) compared to females. There was a trend for a greater increase in Pax7 and CCL2 gene expression in males compared to females (*p* = 0.09).

**Conclusion**: We conclude that there are sex-based differences in the myogenic and inflammatory response, where females have a blunted SC and inflammatory response.

## Introduction

Understanding the underpinnings of skeletal muscle repair and regeneration are important in developing therapeutic strategies to maximize muscle mass and optimize overall muscle health. The ability to regenerate muscle relies upon a population of resident muscle stem cells, referred to as satellite cells (SC) (Lepper et al., 2011). Under resting conditions, SCs are in a quiescent state, but in the presence of appropriate cues, they readily activate, proliferate, and terminally differentiate to aid in repair/regeneration (Dumont et al., 2015). The expression of the Pax7 gene is a hallmark indicator of quiescent and activated SC and is essential for regeneration (Lepper et al., 2011). A network of transcription factors–MyoD, Myf5, myogenin, and MRF4—orchestrate the myogenic response, where activated and proliferating SCs highly express MyoD and Myf5, and differentiating SCs, myogenin and MRF4 (Zammit, 2017). The biological cues that activate SC can originate from several sources within the immediate extracellular space: soluble factors in circulation, adjacent muscle fibers, fibroblasts, endothelial cells, and leukocytes, to name just a few (Chen and Shan, 2019).

When skeletal muscle is damaged, inflammatory cells invade the site of injury within 1–6 h (Tidball, 2005). Neutrophils appear first and produce an oxidative burst (inducing further cellular damage), phagocytose cellular debris, and release cytokines and chemokines to recruit additional inflammatory cells (Tidball, 2005). Macrophage infiltration occurs later, peaking after 48 h following injury, and through the release of cytokines, mediate a shift from a pro- to anti-inflammatory state–a phenomenon critical for muscle repair/regeneration (Peake et al., 2017). Macrophages have classically been defined as type I (M1) and type II (M2); however, they are now commonly characterized as existing on a spectrum, where the pro (M1)- or anti (M2)- inflammatory cell phenotype is designated by the expression of various markers (Xue et al., 2014). Importantly, several other cell types contribute to regeneration such as mast cells, T lymphocytes, eosinophils, fibro-adipogenic progenitors, and pericytes (Chazaud, 2016). The relationship between inflammation and myogenesis is becoming clearer; studies show that macrophages directly influence SC behavior and overall regeneration, which have been comprehensively reviewed elsewhere (Dort et al., 20192019).

Several human studies (MacIntyre et al., 2000; Stupka et al., 2000; Stupka et al., 2001), but not all (Sorichter et al., 2001), report that there are sex-based differences in response to exercise-induced muscle damage. Compared to males, females may be relatively protected against muscle damage, which may be attributed to higher concentrations of estradiol (Dieli-Conwright et al., 2009; Kitajima and Ono, 2016; Le et al., 2018; Collins et al., 2019; Larson et al., 2020). The main mechanisms believed to be responsible for estradiol’s protective effects include: 1) intercalating into the cell membrane affecting fluidity/stability, 2) indirect antioxidant capacity of estradiol to reduce oxidative stress, 3) blunting of the inflammatory response, and/or 4) enhanced expansion of SC. To date, there is no consensus in the literature on the underlying mechanisms of sex-based differences in response to exercise-induced muscle damage. Only three human studies have compared males and females following muscle injury (MacIntyre et al., 2000; Stupka et al., 2000; Stupka et al., 2001), and of these, none reported on the myogenic response. Therefore, the primary objective of the present study was to explore the differences in the myogenic and inflammatory response following exercise-induced muscle damage, in males and females. We hypothesize that 48 h following muscle damage females will have a blunted myogenic and inflammatory response compared with males.

## Materials and Methods

### Participants

Twenty-six healthy young males and females (13M/13F) (See Table 1 for participant characteristics) were recruited to participate in this study. This project was part of a larger study investigating the effects of a multi-ingredient supplement on resistance training outcomes. The study was approved by the Hamilton Health Sciences Integrated Research Ethics Board (Reference No. 4449) and was registered at https://clinicaltrials.gov/as NCT03525197. All participants were recreationally active with no structured exercise in the previous year. Exclusion criteria included smoking, diabetes, the use of nonsteroidal anti-inflammatory drugs (NSAIDs), and/or statins, and history of respiratory disease and/or any major orthopaedic disability. Eleven of the female subjects recruited were normally menstruating, and two were using second-generation oral contraceptives. The study conformed to the guidelines outlined in the Declaration of Helsinki. The participants gave their informed written consent before their inclusion in the study.

**TABLE 1:** Participants’ baseline characteristics.

	Males	Females
Age	21.8 ± 0.6	22.1 ± 0.6
Height (cm)	178.9 ± 1.9	162.4 ± 2.0^a^
Mass (kg)	74.7 ± 1.7	62.4 ± 2.9^a^
Fat mass (kg)	17.4 ± 1.3	20.7 ± 2.0
Lean Mass (kg)	54.2 ± 1.3	39.3 ± 1.4^a^
Leg Lean Mass (kg)	9.6 ± 2.2	6.5 ± 5.6^a^
Body fat %	24.2 ± 1.6	33.7 ± 2.0^a^
1RM Leg Press (lbs)	453.5 ± 37.4	254.6 ± 20.7^a^
1RM Knee Extension (Nm)	188 ± 16.4	105.5 ± 12.5^a^

Values are means ± SEM; *n* = 13 per group. 1-RM one-repetition max.

a Significantly different compared to men.

### Experimental Outline

On the initial assessment day, participants visited the laboratory at approximately 0800, where anthropometric measurements (weight, height), body composition [dual-energy X-ray absorptiometry (DXA) scan], and a muscle biopsy were taken. Participants were then given a brief period of rest before performing 300 maximal eccentric contractions on the biopsied leg, a protocol often used by our laboratory, known to induce damage in the muscle (Nederveen et al., 2018; McKay et al., 2010; O'Reilly et al., 2008). 48 h following the damaging exercise bout, participants visited the laboratory, fasted, to have a second muscle biopsy taken from the same leg.

### Eccentric Damage

Unaccustomed eccentric exercise is a common technique that reliably induces a significant level of skeletal muscle damage, evidenced by extensive z-band streaming, desmin disruption (Proske and Morgan, 2001; Beaton et al., 2002), a significant increase in plasma creatine kinase (Farup et al., 2014), decreased force production (Farup et al., 2014), and upregulation of myogenic regulatory factors (McKay et al., 2008). Participants underwent a brief familiarization session using the Biodex dynamometer (Biodex-System 3, Biodex Medical Systems, Inc., United States) to understand the motion requirements and record relevant apparatus measurements (e.g. seat height, arm length, etc.). Movement at the shoulders, hips, and thigh was restrained with straps, and subjects crossed their arms over their chest to minimize the use of muscles other than the knee extensors. Subjects performed 30 sets of 10 maximal muscle-lengthening contractions with 60 s of rest between sets for 300 lengthening contractions. During each set, investigators provided verbal encouragement to elicit a maximal effort.

### Body Composition

Whole-body lean soft tissue mass (fat-free and bone-free mass), leg lean mass, fat mass, body fat percentage, and bone mineral content were measured (GE Medical Systems Lunar, Madison, WI) and analyzed (Lunar enCORE version 14.1, GE Medical Systems) using a DXA scanner, after a 10- to 12 h overnight fast.

### Muscle Biopsy Sampling

Two muscle biopsies were taken from the mid-portion of the vastus lateralis under local anaesthetic (1% lidocaine) using a 5 mm Bergstrom needle adapted for manual suction. Both biopsies were performed on a single leg, which was randomized for each participant. One muscle biopsy was obtained at rest (pre) and the other 48 h following eccentric damage (post). Approximately 150 mg of muscle tissue was collected from each biopsy. Following collection of the muscle sample, the muscle was dissected free of adipose and connective tissue and flash-frozen in liquid nitrogen, then stored at −80 °C for later analysis. For immunohistochemistry, a fresh piece of muscle (approximately 40 mg) was sectioned from the biopsies, orientated in cross-section, mounted in OCT compound (Tissue-Tek, Sakura Finetek, United States), and frozen in isopentane cooled with liquid nitrogen. The embedded samples were stored at −80°C and then sectioned (7 μm) at −20°C using a cryostat microtome. The cross-sections were mounted on slides and stored at −80°C for immunohistochemical analysis.

### Immunohistochemistry

Muscle cross-sections (7 µm) were prepared from tissue embedded in optimal cutting temperature compound, air-dried for 30 min, and stored at −80°C. Two immunohistochemical experiments were completed, to analyze SC content for type I and II fibers, and to investigate SC activation. Slides were stained with antibodies against Pax7 (neat; Developmental Studies Hybridoma Bank), MyoD (anti-MyoD1; clone 5.8A, 1:100; Dako), A4.951 [myosin heavy chain (MHC) type I, slow isoform, 1:1; Developmental Studies Hybridoma Bank], and MHC-II (fast isoform, 1:1,000; ab91506; Abcam). Secondary antibodies used were Pax7 (Alexa Fluor 488 or 594, 1:500; Invitrogen, Molecular Probes), MyoD (biotinylated secondary antibody, 1:200; Vector Canada; and streptavidin-594 fluorochrome, 1:200; Invitrogen, Molecular Probes), A4.951 (Alexa Fluor 488, 1:500), MHC-II (Alexa Fluor 647, 1:500), and laminin (Wheat Germ Agglutinin 488 or 647, 1:200; Vector Laboratories). Nuclei were labelled with 4,6-diamidino-2-phenylindole (DAPI; 1:20,000; Sigma- Aldrich) before applying a coverslip with fluorescent mounting media (Dako). Slides were viewed with a Nikon Eclipse Ti Microscope (Nikon Instruments), equipped with a high-resolution Photometrics CoolSNAP HQ2 fluorescent camera (Nikon Instruments). Images were captured and analyzed by using Nikon NIS Elements AR 3.2 software (Nikon Instruments). All images were captured with the 20x objective, and ∼249 fibers per participant per time point were included in the analyses for SC content and activation status. The activation status of satellite cells was determined via the colocalization of Pax7, MyoD, and DAPI (i.e., Pax7+/MyoD+). Slides were blinded for both group and time point. All immunofluorescent analyses were completed in a blinded fashion by a single examiner.

### RNA Isolation

Muscle samples were homogenized with 1 ml of TRIzol Reagent (Life Technologies, Burlington, ON, Canada), in Lysing Maxtrix D tubes (MP Biomedicals, Solon, OH), with the FastPrep-24 Tissue and Cell Homogenizer (MP Biomedicals) for a duration of 40 s, at 6 m/s. Following centrifugation, samples were incubated for 5 min to permit complete disassociation of the nucleoprotein complex. Then, 200 µl of chloroform (Sigma-Aldrich, Oakville, ON, Canada) was added, mixed for 10 s, incubated at room temperature for 3 min, and centrifuged for 15 min at 12,000 g at 4°C. The aqueous phase was transferred into a new tube. RNA was precipitated by adding 500 µl of isopropanol (Sigma-Aldrich, Oakville, ON, Canada), incubated at room temperature for 10 min, and centrifuged for 10 min at 12,000 g at 4°C. The resultant RNA pellet was resuspended and washed in 1 ml of 75% ethanol (Commercial Alcohols, Brampton, ON, Canada), centrifuged for 5 min at 7,500 g at 4°C, and the supernatant was discarded. Finally, the RNA pellet was resuspended and diluted in 50 µl of RNase-free water. RNA concentration and purity (260/280) were determined with a Nano-Drop 1,000 Spectrophotometer (Thermo Fisher Scientific, Rockville, MD, United States). The average RNA purity (260/280) was 1.8 ± 0.1(SEM).

### Reverse Transcription

Samples were reverse transcribed using a high-capacity cDNA reverse transcription kit (Applied Biosystems, Foster City, CA, United States), 1000 ng of RNA was diluted in 20 μl reaction solution, as per manufacturer’s instructions. SimpliAmp Thermal Cycler (Thermo Fisher Scientific) was used to obtain cDNA for gene expression analysis.

### Quantitative Real-Time RT-PCR

Quantitative PCR using TaqMan methodology was performed to analyze myogenic (Pax7 [Hs00242962_m1], MyoD [Hs00159528_m1], Myf5 [Hs00929416_g1]), inflammatory (CXCL8 [Hs00174103_m1], CCL2 [Hs00234140_m1], IL-6[Hs00174131_m1], TNFα [Hs02621508_s1]), and pan-macrophage cell surface marker (ITGAM/CD11b [Hs00167304_m1]) gene expression. GAPDH (Hs02786624_g1) was used as the reference gene. For each assay, qPCR was performed using 50 ng of cDNA in TaqMan Fast-Advanced master mix on a QuantStudio 5 (Thermo Fisher Scientific) Real-Time PCR system; samples were run in duplicate. mRNA expression was calculated by using the 2^−ΔΔ^C_t_ method and expressed as fold change from Pre. Due to technical difficulties, only an N = 8 was included for males for CXCL8 gene expression analysis.

### Statistical Analyses

A sample size estimation was conducted (G*power, sample size estimator 3.1.9.4; Kiel, Germany) using the following parameters: average effect size of 0.546 on SC content (Damas et al., 2018), alpha level of 0.05, and minimal power of 0.8, which revealed a minimum sample size of 34 participants. All data are expressed as means ± SEM. Independent samples Student’s *t-tests* were used to compare differences in baseline characteristics between groups. SC content and activation data were analyzed using a two-way repeated-measures ANOVA with biological sex (males and females) as the between-subject factor and time (pre and post) as within-subject factors. Separate ANOVAs were performed for type I, type II, and total fibers. Where significance was found, a Tukey’s post hoc test was performed. Expression of mRNA was analyzed using paired (within-group [time]) or unpaired *t*-test’s (between-group). Analyses were conducted using SPSS statistical package version 23 (IBM SPSS Statistics for Mac, Version 23.0, Armonk, NY).

## Results

### Anthropometrics and Muscle Characteristics

Anthropometric measures at baseline in males and females are reported in Table 1. Males were significantly taller, heavier, and had lower % body fat, compared to females (Table 1, *p* < 0.05). Also, males had greater lean mass and leg lean mass, assessed via DXA (Table 1, *p* < 0.05).

### Satellite Cell Content

**FIGURE 1:**
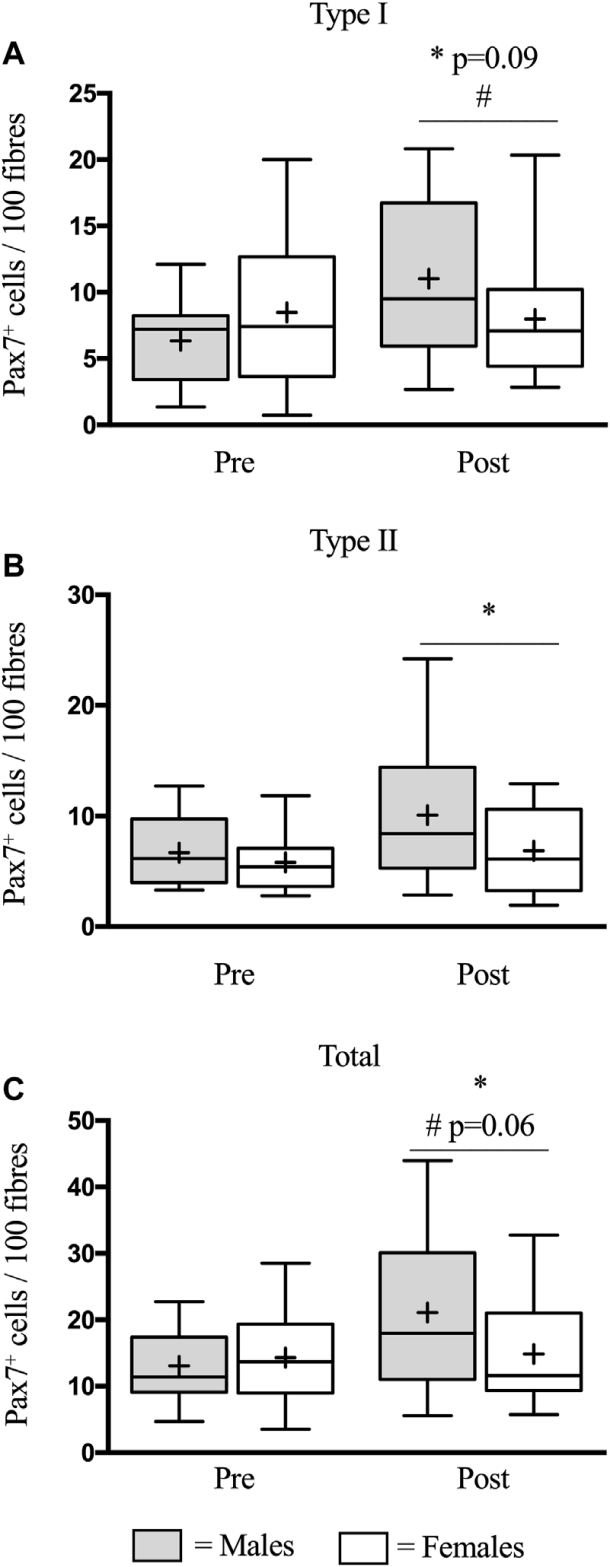
Mean number of satellite cells (SCs) per muscle fibre in type I **(A)**, type II **(B)**, and total **(C)** muscle fibres before (pre) and 48 h (post) following eccentric damage. The box-and-whisker plot displays the median (line) and the mean (+), with the box representing the interquartile range (IQR), and the whiskers representing the maximum and minimum values. ^*^ indicates a significant effect of time (*p* < 0.05). ^#^ indicates time × group interaction.

**FIGURE 2:**
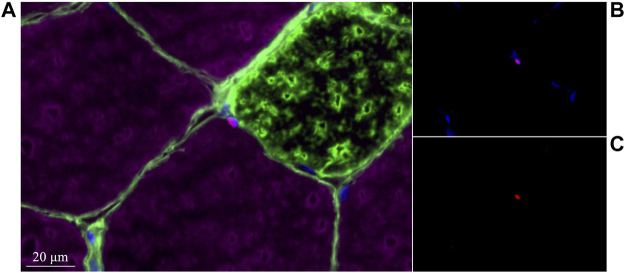
Representative image of a type II-associated satellite cell in the present study. **(A)** MHCI (green) +Laminin (green) +MHCII (purple) +Pax7 (red) +DAPI (blue). **(B)** Pax7 (red) +DAPI (blue). **(C)** Pax7 (red).

In type I fibers, there was a trend (*p* = 0.09) for an increase in SC content from pre-to-post eccentric damage (Figure 1A). We observed a time × group interaction (*p* < 0.05) for type I SC content, where males increased in SC content (6.4 ± 0.8 to 11.0 ± 1.7 Pax7^+^ cells/100 fibers) compared to females (8.5 ± 1.5 to 8.0 ± 1.3 Pax7^+^ cells/100 fibers), following damage (Figure 1A). In type II fibers, there was an increase (*p* < 0.05) in SC content from pre-to-post, but no group differences (Figure 1B). A representative image of a type II fiber-associated SC is seen in Figure 2. In total fibers–collapsed type I and type II fibers–we report an effect of time (*p* < 0.05) (Figure 1C). Additionally, there was a strong trend for a time × group interaction (*p* = 0.06); males demonstrated a larger increase in total SC (13.0 ± 1.5 to 21.1 ± 3.2 Pax7^+^ cells/100 fibers), compared to females (14.3 ± 2.1 to 14.9 ± 2.2 Pax7^+^ cells/100 fibers) (Figure 1C).

### Satellite Cell Activation

**FIGURE 3:**
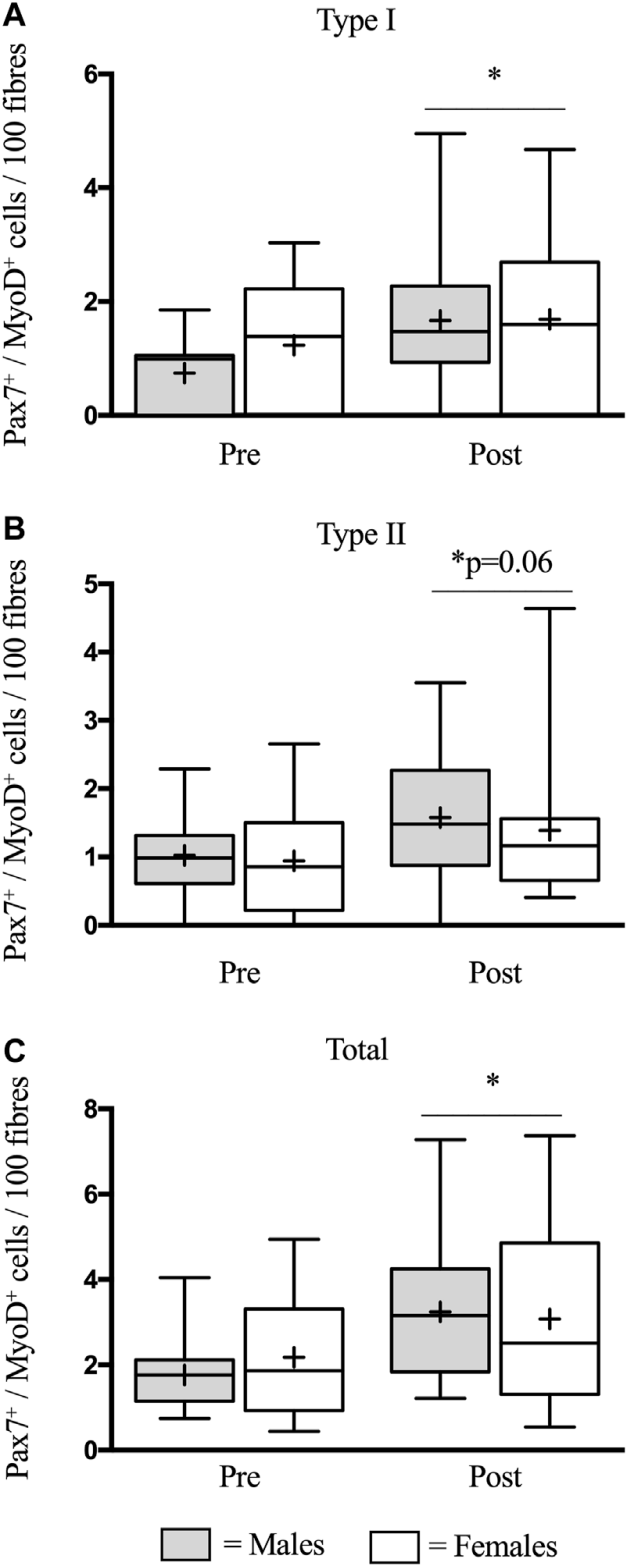
Mean number of activated satellite cells (SCs) per muscle fibre in type I **(A)**, type II **(B)**, and total **(C)** muscle fibres before (pre) and 48 h (post) following eccentric damage. The box-and-whisker plot displays the median (line) and the mean (+), with the box representing the interquartile range (IQR), and the whiskers representing the maximum and minimum values. ^*^ indicates a significant effect of time (*p* < 0.05).

**FIGURE 4:**
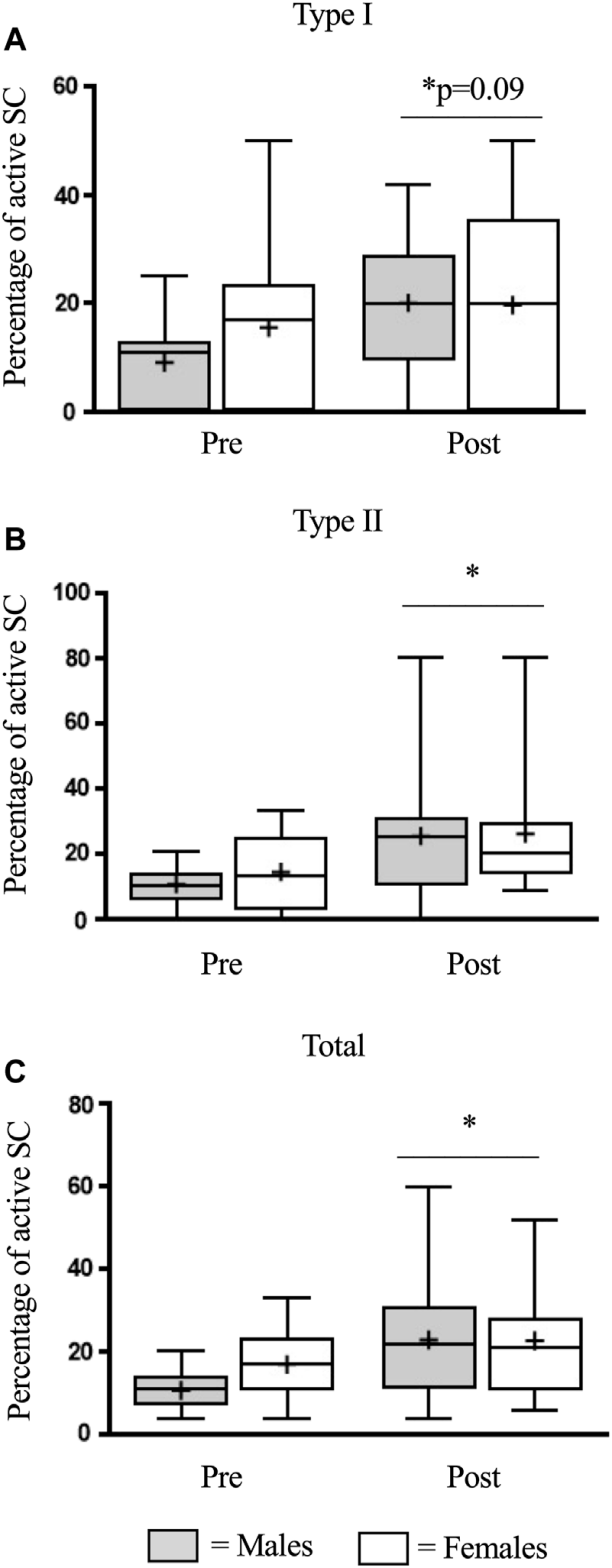
The percentage of activated satellite cells (SC) in type I **(A)**, type II **(B)**, and total **(C)** muscle fibres before (pre) and 48 h (post) following eccentric damage. The box-and-whisker plot displays the median (line) and the mean (+), with the box representing the interquartile range (IQR), and the whiskers representing the maximum and minimum values. ^*^ indicates a significant effect of time (*p* < 0.05).

In type I fibers, there was a similar increase (*p* < 0.05) in SC activation in males (0.7 ± 0.2 to 1.7 ± 0.3 Pax7^+^/MyoD^+^ cells/100 fibers) and females (1.2 ± 0.3 to 1.7 ± 0.4 Pax7^+^/MyoD^+^ cells/100 fibers) following eccentric damage (*p* < 0.05) (Figure 3A). Likewise, in type II fibers, there was a strong trend for an increase (*p* = 0.06) in SC activation in males (1.0 ± 0.2 to 1.6 ± 0.3 Pax7^+^/MyoD^+^ cells/100 fibers) and females (0.9 ± 0.2 to 1.40 ± 0.3 Pax7^+^/MyoD^+^ cells/100 fibers) following eccentric damage (Figure 3B). Finally, there was an increase (*p* < 0.05) in total SC activation in males (1.8 ± 0.2 to 3.2 ± 0.5 Pax7^+^/MyoD^+^ cells/100 fibers) and females (2.2 ± 0.4 to 3.1 ± 0.6 Pax7^+^/MyoD^+^ cells/100 fibers) following eccentric damage (*p* < 0.05) (Figure 3C). The proportion of active SCs were expressed as active SC relative to total SC abundance. In type I fibers, there was a trend for an increase (*p* = 0.09) in the proportion of active SC in males (9 ± 2%–20 ± 4%) and females (16 ± 4%–20 ± 5%) (Figure 4A) but no differences were detected between groups. Comparably, in type II fibers, there was a similar increase (*p* < 0.05) in the proportion of active SC in males (11 ± 2%–25 ± 6%) and females (14 ± 3%–26 ± 6%) (Figure 4B). In total fibers, males (10 ± 1%–23 ± 4%) and females (17 ± 2%–23 ± 4%) increased (*p* < 0.05) but no differences were found between groups (Figure 4C).

### Myogenic Gene Expression

**FIGURE 5:**
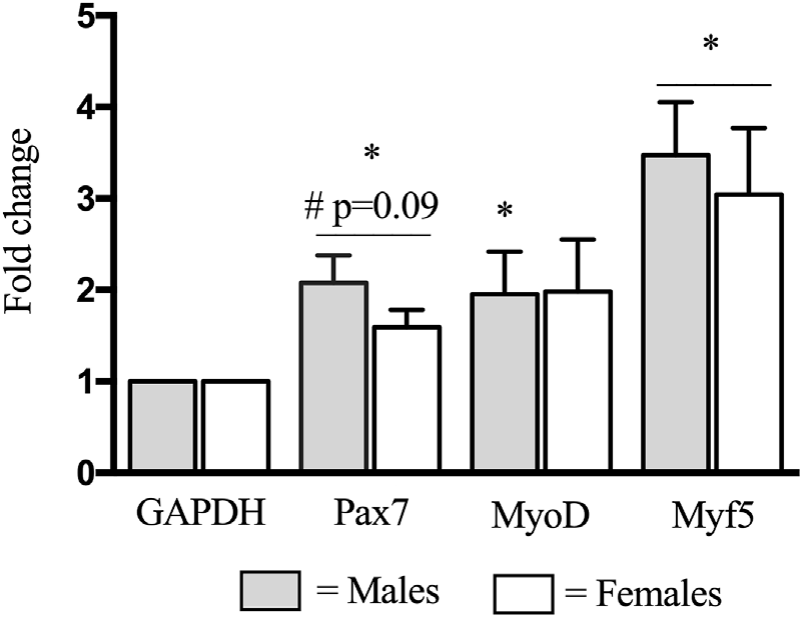
Changes in Pax7, MyoD, and Myf5 mRNA expression before (pre-) and 48 h (post) following eccentric damage. Fold changes for mRNA expression were calculated using the 2^−ΔΔ^C_t_ method with genes of interest normalized to GAPDH. ^*^ indicates significant effect of time (*p* < 0.05). ^#^ indicates significant time × group interaction; an increase from pre-exercise value is smaller in females compared with males (*p* < 0.05).

Prior to the bout of damaging exercise, there was no significant difference between males and females in myogenic gene expression (*p* > 0.05). Fold changes for mRNA expression are presented with genes of interest normalized to GAPDH. In response to eccentric damage, there was a trend (*p* = 0.09) for a time × group interaction for Pax7 gene expression, where males (2.1-fold change) had a higher expression, compared to females (1.6-fold change) (Figure 5). Both MyoD and Myf5 expression increased (*p* < 0.05) similarly in males (MyoD: 2.0-fold change; Myf5: 3.5-fold change) and females (MyoD: 2.0-fold change; Myf5: 3.0-fold change) (Figure 5).

### Inflammatory Gene Expression

**FIGURE 6:**
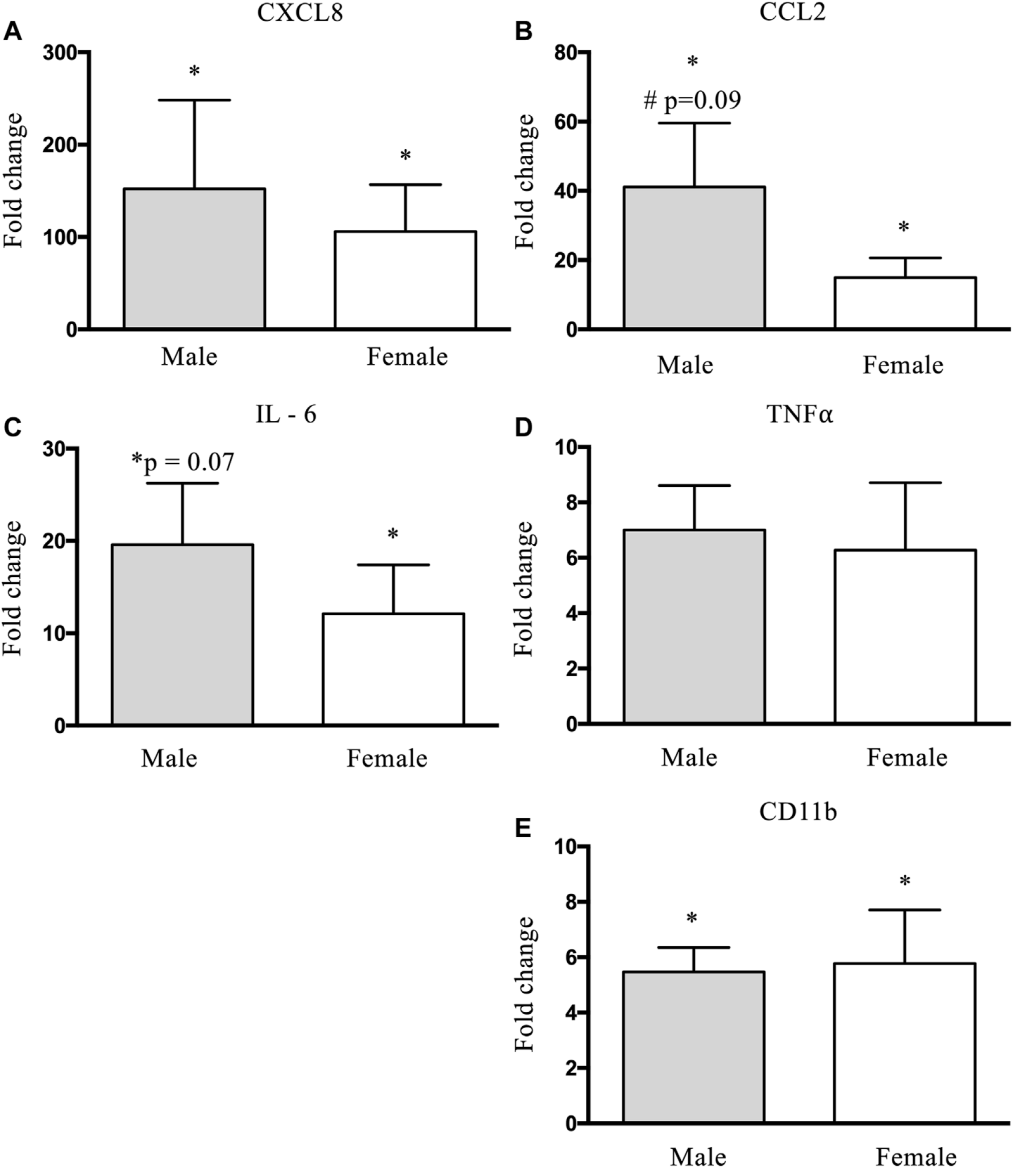
Changes in CXCL8 **(A)**, CCL2 **(B)**, IL-6 **(C)**, TNFα **(D)**, and CD11b **(E)** mRNA expression before (pre-) and 48 h (post) following eccentric damage. Fold changes for mRNA expression were calculated using the 2^−ΔΔ^C_t_ method with genes of interest normalized to GAPDH. ^*^ indicates significant effect of time (*p* < 0.05). ^#^ indicates significant time × group interaction; an increase from pre-exercise value is smaller in females compared with males (*p* < 0.05).

Prior to the bout of damaging exercise, there was no significant difference between males and females in inflammatory gene expression (*p* > 0.05). There was an increase (*p* < 0.05) in CXCL8 expression in males (152.2-fold change) and females (105.9-fold change) (Figure 6A). There was a trend (*p* = 0.09) for a time × group interaction for CCL2 expression, where males had a larger (41.1-fold change) increase than females (15.0-fold change) (Figure 6B). IL6 increased (*p* < 0.05) in females (12.1-fold change) and showed a trend (*p* = 0.07) for an increase in males (19.6-fold change; Figure 6C). TNFα did not significantly increase in males or females (Figure 6D). CD11b increased (*p* < 0.05) in males (5.5-fold change) and females (5.8-fold change) to a similar extent (Figure 6E).

## Discussion

The major finding from this study was that following 300 maximal eccentric contractions, males had a significantly greater increase in SC expansion compared to females. Furthermore, we observed a trend for a greater increase in Pax7 and CCL2 expression in males 48 h following muscle damage compared to females. To our knowledge, this is the first investigation to report a sex-based difference in the SC response, following muscle damage in humans.

Most human studies investigating the SC response, *in vivo,* have been conducted in males only. In this study, males experienced a 60% increase (*p* < 0.05) in total SC content following damage, which is similar to what we have previously reported–in males–using this protocol (Nederveen et al., 2018; McKay et al., 2010; O'Reilly et al., 2008) (Figure 1C). The response in mixed fiber SC content was primarily driven by type I fiber-associated SC, such that a time × group interaction was detected in type I, but not type II fibers. This finding was surprising, as studies in young males consistently report a preponderant increase in type II fiber-associated SC, following maximal eccentric contractions (Cermak et al., 2013; Farup et al., 2014; Nederveen et al., 2018). In our study, type II SC in males increased ∼3.2 times more than females, however, due to variability in the dataset we did not detect a significant result. Strikingly, in females, there was a complete absence of SC expansion (Figure 1C) following damage. In line with this, males had a two times greater proportion of active type I fiber-associated SC (as a proportion of total SC) compared to females. Importantly, this was not statistically significant at *p* < 0.05 (*p* = 0.11), nonetheless, this finding is consistent with the greater expansion of SC observed in males. This differential SC response is further corroborated through gene expression analysis, where a trend was detected for greater Pax7 mRNA expression in males, compared to females (Figure 5). Together, our immunohistochemical and gene expression analysis suggests that males readily mobilize SC following contraction-induced damage, whereas females do not or are delayed in doing so.

We sought to determine if there was an altered inflammatory gene expression response between males and females. Since M1-macrophage abundance is elevated at 48 h (Peake et al., 2017), we focused our analysis on pro-inflammatory cytokines/chemokines that have been shown to increase with exercise and influence SC behaviour: chemokine ligand 8 (CXCL8/IL-8) (Nieman et al., 2004), chemokine ligand 2 (CCL2) (Hubal et al., 2010), interleukin 6 (IL-6) (Muñoz‐Cánoves et al., 2013), and tumour necrosis factor-alpha (TNFα) (Crane et al., 2012). CCL2 helps promote the recruitment and differentiation of Ly6C+ monocytes to M1 macrophages as part of the proinflammatory cascade (Jetten et al., 2014). IL-8 induces macrophage infiltration to the lesion site (Fujishima et al., 1993). Macrophages then release TNF-a and IL-6, attracting stem cells to the site of damage and stimulating myoblast proliferation and differentiation (Muñoz‐Cánoves et al., 2013). Males trended to have a higher CCL2 mRNA expression compared to females following damage (Figure 6B). CCL2 is a ligand that binds to chemokine receptor 2 (CCR2), and this CCL2-CCR2 axis is the main entry point during monocyte invasion into the injured cell (Chazaud, 2016). Additionally, culturing CCL2 with C2C12 myoblasts increases phosphorylation of extracellular signal-regulated kinase (ERK)1/2 and mitogen-activated protein kinase (MAPK) and enhances cell proliferation (Yahiaoui et al., 2008). This cascade appears to be a key modulator, as disruption of the CCL2-CCR2 axis alters inflammation and impairs muscle regeneration (Shireman et al., 2007). Thus, considering the proliferative effects of CCL2, it is reasonable to speculate that the increased CCL2 expression in males may have contributed to the larger increase in SC content. Though no group differences were detected, when collapsed, all other cytokines increased or trended to increase (Figures 6A,C), following damage, except TNFα (Figure 6D). To gain insight into the macrophage response, we analyzed CD11b mRNA expression and observed similar increases in males and females (Figure 6E). Assuming similar translational capacity between sexes, it would be reasonable to speculate that males and females would also express similar increases in macrophage cell content in the muscle 48 h post-exercise. Together, our findings suggest a sex-based difference in the myogenic response to damage and evidence that the inflammatory response following eccentric damage may be different between males and females.

The present observations may reflect underlying sex-based differences in muscle damage after the same relative load of exercise, as has been indicated in previous animal (Komulainen et al., 1999; Tiidus and Bombardier, 1999; Sorichter et al., 2001; Lu et al., 2011) and human (MacIntyre et al., 2000; Stupka et al., 2000; Stupka et al., 2001) literature. Following damage, female rodents exhibit less structural disorganization and necrosis as compared to male rodents (Komulainen et al., 1999; Tiidus and Bombardier, 1999). Furthermore, animal studies indicate greater expression of CCL2 in damaged myofibers (Lu et al., 2011), an observation we found to be greater in males in the current investigation, possibly reflecting a greater degree of muscle damage. Human studies also indicate less z-disk streaming and inflammation in females as compared to males after damaging exercise (MacIntyre et al., 2000; Stupka et al., 2000; Stupka et al., 2001). The current study presents novel findings on sex-based differences in the myogenic response to the same relative load of damaging exercise, adding to the existing literature on damaging exercise and highlighting a potential mechanistic link between satellite cells and the muscle damage response in males and females.

A notable difference between males and females that could influence muscle repair is the difference in sex hormone profile, specifically a higher concentration of testosterone in males and estrogens in females. While much of testosterone’s effects on muscle inflammation and regeneration have been described (Velders and Diel, 2013), little is known regarding the effects of estrogens. Most studies manipulate estradiol and observe the impact on the inflammatory response. This approach has yielded mixed findings, where higher estradiol concentrations are shown to reduce (St. Pierre Schneider et al., 1999; Tiidus and Bombardier, 1999; Stupka and Tiidus, 2001; Tiidus et al., 2001; Tiidus et al., 2005a; Enns et al., 2008; Iqbal et al., 2008), increase (Tiidus et al., 2005b; St. Pierre Schneider et al., 2012; Fulkerson et al., 2015; Fearing et al., 2016; Le et al., 2018), or have no effect on the inflammatory response (Schneider et al., 2002; McHale et al., 2012; Velders et al., 2012). The discrepancy in these findings is likely attributable to the model of damage, the method of estrogen depletion in controls, the species studied, and the variable levels of estradiol used in the experimental groups (many studies do not report the estrogen concentrations). Adding to the complexity, estrogen receptors, alpha (Collins et al., 2018), and beta (Seko et al., 2020), are both essential for muscle regeneration and their abundance appears to vary throughout the menstrual cycle (Ekenros et al., 2017). Although the research provides valuable insight on estrogen’s potential protective effect against muscle damage, it does not directly compare males and females. Of the three studies conducted in humans directly comparing males and females following skeletal muscle damage, none reported on SC content, or any markers of myogenesis (MacIntyre et al., 2000; Stupka et al., 2000; Stupka et al., 2001); thus, our finding of a differential SC response is novel.

This study was not without limitations. Firstly, we did not have access to serum samples, and we did not record the menstrual phase during the experimental protocol, thus we were unable to measure or estimate estrogen concentration at the time of the biopsies. However, it is important to recognize that regardless of menstrual cycle phase, females would have had a significantly higher concentration of estrogen compared to males. Second, we were limited to one timepoint following eccentric damage, more timepoints would have permitted a more comprehensive investigation of the early- and late-myogenic and inflammatory responses. Third, we were unable to provide any traditional markers of muscle damage such as creatine kinase serum concentration, a decline in muscle strength, or Z-line streaming due to a lack of tissue availability. Despite these limitations, our results demonstrate that SC expansion is different between males and females following the same relative eccentric-focused bout of muscle damage. Furthermore, the inflammatory response in females, as characterized through gene expression responses was significantly different to that of males. To further reveal sex-based differences in muscle regeneration/repair in humans, future studies should focus on comparing males and females, while accounting for serum estrogen concentration and/or menstrual phase at the time of biopsy. Moreover, an extensive post-damage time-course will allow for more extensive characterization of the inflammatory and regenerative/repair response.

## Data Availability

The original contributions presented in the study are included in the article/Supplementary Materials, further inquiries can be directed to the corresponding author.

## References

[B1] Beaton L. J. Tarnopolsky M. A. Phillips S. M. (2002). Variability in Estimating Eccentric Contraction-Induced Muscle Damage and Inflammation in Humans. Can. J. Appl. Physiol. 27, 516–526. 10.1139/h02-028 12429897

[B2] Cermak N. M. Snijders T. McKay B. R. Parise G. Verdijk L. B. Tarnopolsky M. A. (2013). Eccentric Exercise Increases Satellite Cell Content in Type II Muscle Fibers. Med. Sci. Sports Exerc. 45, 230–237. 10.1249/MSS.0b013e318272cf47 22968308

[B3] Chazaud B. (2016). Inflammation during Skeletal Muscle Regeneration and Tissue Remodeling: Application to Exercise‐induced Muscle Damage Management. Immunol. Cel Biol 94, 140–145. 10.1038/icb.2015.97 26526620

[B4] Chen B. Shan T. (2019). The Role of Satellite and Other Functional Cell Types in Muscle Repair and Regeneration. J. Muscle Res. Cel Motil 40, 1–8. 10.1007/s10974-019-09511-3 30968305

[B5] Collins B. C. Arpke R. W. Larson A. A. Baumann C. W. Xie N. Cabelka C. A. (2019). Estrogen Regulates the Satellite Cell Compartment in Females. Cel Rep. 28, 368–381. 10.1016/j.celrep.2019.06.025 PMC6655560 31291574

[B6] Collins B. C. Mader T. L. Cabelka C. A. Iñigo M. R. Spangenburg E. E. Lowe D. A. (2018). Deletion of Estrogen Receptor α in Skeletal Muscle Results in Impaired Contractility in Female Mice. J. Appl. Physiol. 124, 980–992. 10.1152/japplphysiol.00864.2017 29345963 PMC5972463

[B7] Crane J. D. Ogborn D. I. Cupido C. Melov S. Hubbard A. Bourgeois J. M. (2012). Massage Therapy Attenuates Inflammatory Signaling after Exercise-Induced Muscle Damage. Sci. Transl. Med. 4, 4. 10.1126/scitranslmed.3002882 22301554

[B8] Damas F. Libardi C. A. Ugrinowitsch C. Vechin F. C. Lixandrão M. E. Snijders T. (2018). Early- and Later-Phases Satellite Cell Responses and Myonuclear Content with Resistance Training in Young Men. PLoS ONE 13, e0191039. 10.1371/journal.pone.0191039 29324825 PMC5764368

[B9] Dieli-Conwright C. M. Spektor T. M. Rice J. C. Schroeder E. T. (2009). Hormone Therapy Attenuates Exercise-Induced Skeletal Muscle Damage in Postmenopausal Women. J. Appl. Physiol. 107, 853–858. 10.1152/japplphysiol.00404.2009 19574506 PMC4073923

[B10] Dort J. Fabre P. Molina T. Dumont N. A. (20192019). Macrophages Are Key Regulators of Stem Cells during Skeletal Muscle Regeneration and Diseases. Stem Cell Int. 2019, 1–20. 10.1155/2019/4761427 PMC6664695 31396285

[B11] Dumont N. A. Wang Y. X. Rudnicki M. A. (2015). Intrinsic and Extrinsic Mechanisms Regulating Satellite Cell Function. Development 142, 1572–1581. 10.1242/dev.114223 25922523 PMC4419274

[B12] Ekenros L. Papoutsi Z. Fridén C. Dahlman Wright K. Lindén Hirschberg A. (2017). Expression of Sex Steroid Hormone Receptors in Human Skeletal Muscle during the Menstrual Cycle. Acta Physiol. 219, 486–493. 10.1111/apha.12757 27438889

[B13] Enns D. L. Iqbal S. Tiidus P. M. (2008). Oestrogen Receptors Mediate Oestrogen-Induced Increases in post-exercise Rat Skeletal Muscle Satellite Cells. Acta Physiol. 194, 81–93. 10.1111/j.1748-1716.2008.01861.x 18397384

[B14] Farup J. Rahbek S. K. Knudsen I. S. de Paoli F. Mackey A. L. Vissing K. (2014). Whey Protein Supplementation Accelerates Satellite Cell Proliferation during Recovery from Eccentric Exercise. Amino Acids 46, 2503–2516. 10.1007/s00726-014-1810-3 25063205

[B15] Fearing C. M. Melton D. W. Lei X. Hancock H. Wang H. Sarwar Z. U. (2016). Increased Adipocyte Area in Injured Muscle with Aging and Impaired Remodeling in Female Mice. Gerona 71, 992–1004. 10.1093/gerona/glv104 PMC4945881 26273023

[B16] Fujishima S. Hoffman A. R. Vu T. Kim K. J. Zheng H. Daniel D. (1993). Regulation of Neutrophil Interleukin 8 Gene Expression and Protein Secretion by LPS, TNF-?, and IL-1? J. Cel. Physiol. 154, 478–485. 10.1002/jcp.1041540305 8436597

[B17] Fulkerson N. Nicholas J. St. Pierre Schneider B. B. (2015). Estrogen Modulates 7/4 Antigen Distribution within Eccentrically Contracted Injured Skeletal Muscle. Biotech. Histochem. 90, 294–301. 10.3109/10520295.2014.992961 25747047 PMC5639701

[B18] Hubal M. J. Devaney J. M. Hoffman E. P. Zambraski E. J. Gordish-Dressman H. Kearns A. K. (2010). CCL2 and CCR2 Polymorphisms Are Associated with Markers of Exercise-Induced Skeletal Muscle Damage. J. Appl. Physiol. 108, 1651–1658. 10.1152/japplphysiol.00361.2009 20339010

[B19] Iqbal S. Thomas A. Bunyan K. Tiidus P. M. (2008). Progesterone and Estrogen Influence Postexercise Leukocyte Infiltration in Overiectomized Female Rats. Appl. Physiol. Nutr. Metab. 33, 1207–1212. 10.1139/H08-108 19088779

[B20] Jetten N. Verbruggen S. Gijbels M. J. Post M. J. De Winther M. P. J. Donners M. M. P. C. (2014). Anti-inflammatory M2, but Not Pro-inflammatory M1 Macrophages Promote Angiogenesis *In Vivo* . Angiogenesis 17, 109–118. 10.1007/s10456-013-9381-6 24013945

[B21] Kitajima Y. Ono Y. (2016). Estrogens Maintain Skeletal Muscle and Satellite Cell Functions. J. Endocrinol. 229, 267–275. 10.1530/JOE-15-0476 27048232

[B22] Komulainen J. Koskinen S. O. A. Kalliokoski R. Takala T. E. S. Vihko V. (1999). Gender Differences in Skeletal Muscle Fibre Damage after Eccentrically Biased Downhill Running in Rats. Acta Physiol. Scand. 165, 57–63. 10.1046/j.1365-201x.1999.00481.x 10072098

[B23] Larson A. A. Baumann C. W. Kyba M. Lowe D. A. (2020). Oestradiol Affects Skeletal Muscle Mass, Strength and Satellite Cells Following Repeated Injuries. Exp. Physiol. 105, 1700–1707. 10.1113/EP088827 32851730 PMC7722039

[B24] Le G. Novotny S. A. Mader T. L. Greising S. M. Chan S. S. K. Kyba M. (2018). A Moderate Oestradiol Level Enhances Neutrophil Number and Activity in Muscle after Traumatic Injury but Strength Recovery Is Accelerated. J. Physiol. 596, 4665–4680. 10.1113/JP276432 30035314 PMC6166067

[B25] Lepper C. Partridge T. A. Fan C.-M. (2011). An Absolute Requirement for Pax7-Positive Satellite Cells in Acute Injury-Induced Skeletal Muscle Regeneration. Development 138, 3639–3646. 10.1242/dev.067595 21828092 PMC3152922

[B26] Lu H. Huang D. Ransohoff R. M. Zhou L. (2011). Acute Skeletal Muscle Injury: CCL2 Expression by Both Monocytes and Injured Muscle Is Required for Repair. FASEB j. 25, 3344–3355. 10.1096/fj.10-178939 21697550 PMC3177578

[B27] MacIntyre D. L. Reid W. D. Lyster D. M. McKenzie D. C. (2000). Different Effects of Strenuous Eccentric Exercise on the Accumulation of Neutrophils in Muscle in Women and Men. Eur. J. Appl. Physiol. 81, 47–53. 10.1007/PL00013796 10552266

[B28] McHale M. J. Sarwar Z. U. Cardenas D. P. Porter L. Salinas A. S. Michalek J. E. (2012). Increased Fat Deposition in Injured Skeletal Muscle Is Regulated by Sex-specific Hormones. Am. J. Physiology-Regulatory, Integr. Comp. Physiol. 302, R331–R339. 10.1152/ajpregu.00427.2011 PMC3289262 22116509

[B29] McKay B. R. O'Reilly C. E. Phillips S. M. Tarnopolsky M. A. Parise G. (2008). Co-expression of IGF-1 Family Members with Myogenic Regulatory Factors Following Acute Damaging Muscle-Lengthening Contractions in Humans. J. Physiol. 586, 5549–5560. 10.1113/jphysiol.2008.160176 18818249 PMC2655389

[B30] McKay B. R. Toth K. G. Tarnopolsky M. A. Parise G. (2010). Satellite Cell Number and Cell Cycle Kinetics in Response to Acute Myotrauma in Humans: Immunohistochemistryversusflow Cytometry. J. Physiol. 588, 3307–3320. 10.1113/jphysiol.2010.190876 20624792 PMC2976024

[B31] Muñoz‐Cánoves P. Scheele C. Pedersen B. K. Serrano A. L. (2013). Interleukin‐6 Myokine Signaling in Skeletal Muscle: a Double‐edged Sword? Febs J. 280, 4131–4148. 10.1111/febs.12338 23663276 PMC4163639

[B32] Nederveen J. P. Joanisse S. Snijders T. Thomas A. C. Q. Kumbhare D. Parise G. (2018). The Influence of Capillarization on Satellite Cell Pool Expansion and Activation Following Exercise-Induced Muscle Damage in Healthy Young Men. J. Physiol. 596, 1063–1078. 10.1113/JP275155 29315567 PMC5851891

[B33] Nieman D. C. Davis J. M. Brown V. A. Henson D. A. Dumke C. L. Utter A. C. (2004). Influence of Carbohydrate Ingestion on Immune Changes after 2 H of Intensive Resistance Training. J. Appl. Physiol. 96, 1292–1298. 10.1152/japplphysiol.01064.2003 14672962

[B34] O'Reilly C. McKay B. Phillips S. Tarnopolsky M. Parise G. (2008). Hepatocyte Growth Factor (HGF) and the Satellite Cell Response Following Muscle Lengthening Contractions in Humans. Muscle Nerve 38, 1434–1442. 10.1002/mus.21146 18816607

[B35] Peake J. M. Neubauer O. Della Gatta P. A. Nosaka K. (2017). Muscle Damage and Inflammation during Recovery from Exercise. J. Appl. Physiol. 122, 559–570. 10.1152/japplphysiol.00971.2016 28035017

[B36] Proske U. Morgan D. L. (2001). Muscle Damage from Eccentric Exercise: Mechanism, Mechanical Signs, Adaptation and Clinical Applications. J. Physiol. 537, 333–345. 10.1111/j.1469-7793.2001.00333.x 11731568 PMC2278966

[B37] Schneider B. S. P. Sannes H. Fine J. Best T. (2002). Desmin Characteristics of CD11b-Positive Fibers after Eccentric Contractions. Med. Sci. Sports Exerc. 34, 274–281. 10.1097/00005768-200202000-00015 11828237

[B38] Seko D. Fujita R. Kitajima Y. Nakamura K. Imai Y. Ono Y. (2020). Estrogen Receptor β Controls Muscle Growth and Regeneration in Young Female Mice. Stem Cel Rep. 15, 577–586. 10.1016/j.stemcr.2020.07.017 PMC7486216 32822588

[B39] Shireman P. K. Contreras-Shannon V. Ochoa O. Karia B. P. Michalek J. E. McManus L. M. (2007). MCP-1 Deficiency Causes Altered Inflammation with Impaired Skeletal Muscle Regeneration. J. Leukoc. Biol. 81, 775–785. 10.1189/jlb.0506356 17135576

[B40] Sorichter S. Mair J. Koller A. Calzolari C. Huonker M. Pau B. (2001). Release of Muscle Proteins after Downhill Running in Male and Female Subjects. Scand. J. Med. Sci. Sports 11, 28–32. 10.1034/j.1600-0838.2001.011001028.x 11169232

[B41] St. Pierre Schneider B. Correia L. A. Cannon J. G. (1999). Sex Differences in Leukocyte Invasion in Injured Murine Skeletal Muscle. Res. Nurs. Health, 22. 10.1002/(SICI)1098-240X 10344704

[B42] St. Pierre Schneider B. St. Pierre Schneider B. Vigil S. A. Moonie S. (2012). Body Weight and Leukocyte Infiltration after an Acute Exercise-Related Muscle Injury in Ovariectomized Mice Treated with Estrogen and Progesterone. Gen. Comp. Endocrinol. 176, 144–150. 10.1016/j.ygcen.2011.12.019 22233774 PMC3319700

[B43] Stupka N. Lowther S. Chorneyko K. Bourgeois J. M. Hogben C. Tarnopolsky M. A. (2000). Gender Differences in Muscle Inflammation after Eccentric Exercise. J. Appl. Physiol. 89, 2325–2332. 10.1152/jappl.2000.89.6.2325 11090586

[B44] Stupka N. Tarnopolsky M. A. Yardley N. J. Phillips S. M. (2001). Cellular Adaptation to Repeated Eccentric Exercise-Induced Muscle Damage. J. Appl. Physiol. 91, 1669–1678. 10.1152/jappl.2001.91.4.1669 11568149

[B45] Stupka N. Tiidus P. M. (2001). Effects of Ovariectomy and Estrogen on Ischemia-Reperfusion Injury in Hindlimbs of Female Rats. J. Appl. Physiol. 91, 1828–1835. 10.1152/jappl.2001.91.4.1828 11568169

[B46] Tidball J. G. (2005). Inflammatory Processes in Muscle Injury and Repair. Am. J. Physiology-Regulatory, Integr. Comp. Physiol. 288, R345–R353. 10.1152/ajpregu.00454.2004 15637171

[B47] Tiidus P. M. Bombardier E. (1999). Oestrogen Attenuates post‐exercise Myeloperoxidase Activity in Skeletal Muscle of Male Rats. Acta Physiol. Scand. 166, 85–90. 10.1046/j.1365-201x.1999.00550.x 10383486

[B48] Tiidus P. M. Deller M. Bombardier E. Gül M. Liu X. L. (2005). Estrogen Supplementation Failed to Attenuate Biochemical Indices of Neutrophil Infiltration or Damage in Rat Skeletal Muscles Following Ischemia. Biol. Res. 38, 38. 10.4067/S0716-97602005000200011 16238100

[B49] Tiidus P. M. Deller M. Liu X. L. (2005). Oestrogen Influence on Myogenic Satellite Cells Following Downhill Running in Male Rats: A Preliminary Study. Acta Physiol. Scand. 184, 67–72. 10.1111/j.1365-201X.2005.01427.x 15847645

[B50] Tiidus P. M. Holden D. Bombardier E. Zajchowski S. Enns D. Belcastro A. (2001). Estrogen Effect on post-exercise Skeletal Muscle Neutrophil Infiltration and Calpain Activity. Can. J. Physiol. Pharmacol. 79, 400–406. 10.1139/y01-011 11405243

[B51] Velders M. Diel P. (2013). How Sex Hormones Promote Skeletal Muscle Regeneration. Sports Med. 43, 1089–1100. 10.1007/s40279-013-0081-6 23888432

[B52] Velders M. Schleipen B. Fritzemeier K. H. Zierau O. Diel P. (2012). Selective Estrogen Receptor‐β Activation Stimulates Skeletal Muscle Growth and Regeneration. FASEB j. 26, 1909–1920. 10.1096/fj.11-194779 22278942

[B53] Xue J. Schmidt S. V. Sander J. Draffehn A. Krebs W. Quester I. (2014). Transcriptome-Based Network Analysis Reveals a Spectrum Model of Human Macrophage Activation. Immunity 40, 274–288. 10.1016/j.immuni.2014.01.006 24530056 PMC3991396

[B54] Yahiaoui L. Gvozdic D. Danialou G. Mack M. Petrof B. J. (2008). CC Family Chemokines Directly Regulate Myoblast Responses to Skeletal Muscle Injury. J. Physiol. 586, 3991–4004. 10.1113/jphysiol.2008.152090 18566004 PMC2538927

[B55] Zammit P. S. (2017). Function of the Myogenic Regulatory Factors Myf5, MyoD, Myogenin and MRF4 in Skeletal Muscle, Satellite Cells and Regenerative Myogenesis. Semin. Cel Develop. Biol. 72, 19–32. 10.1016/j.semcdb.2017.11.011 29127046

